# The Impact of Information and Communication Technology Industrial Co-Agglomeration on Carbon Productivity with the Background of the Digital Economy: Empirical Evidence from China

**DOI:** 10.3390/ijerph20010316

**Published:** 2022-12-25

**Authors:** Xiaowen Wang, Nishang Tian, Shuting Wang

**Affiliations:** School of Economics, Lanzhou University, Lanzhou 730000, China

**Keywords:** digital economy, ICT industrial co-agglomeration, carbon productivity, dynamic panel data model, GTWR

## Abstract

In the era of the digital economy, the information and communication technology (ICT) industry has opened up a new round of expansion, while forming co-located development in the space. ICT industrial co-agglomeration has tremendous advantages in promoting economic development and achieving carbon neutrality goals. This paper analyzes the spatio-temporal characteristics of ICT industrial co-agglomeration and carbon productivity from 2009 to 2019 in China. It empirically explores the impact of ICT industrial co-agglomeration on carbon productivity using a systematic GMM model. Additionally, it analyses the spatial and temporal heterogeneity of ICT industrial co-agglomeration and other factors affecting carbon productivity using a geographically and temporally weighted regression (GTWR) model. The findings are as follows: (1) China’s ICT industrial co-agglomeration and carbon productivity show an upward trend. Additionally, their characteristic of regional distribution is east–high and west–low. (2) ICT industrial co-agglomeration has a positive association with carbon productivity. (3) The impact of ICT industrial co-agglomeration on carbon productivity has significant spatial and temporal heterogeneity. The regression coefficient of ICT industrial co-agglomeration increases continuously during the study period, and the degree of impact is relatively larger in Northern China. As the degree of ICT industrial co-agglomeration continues to increase, its positive impact on carbon productivity across China is deepening. The findings of this paper complete the research on the impact of ICT industrial co-agglomeration on carbon productivity, and the related policy recommendations provide useful references for the digital economy and sustainable development.

## 1. Introduction

The problem of global warming caused by greenhouse gas emissions has become one of the important factors limiting the sustainable development of human society. With the accelerated pace of industrialization and urbanization, the demand for energy consumption and carbon emissions continues to grow, and global climate problems are frequent, with green development gradually becoming an international consensus [1,2]. With the dilemma of carbon emission reduction and economic growth being mutually constrained, countries worldwide are actively transforming their economic development patterns to achieve green, low-carbon and sustainable economic goals to improve the “extensive economy growth” at the cost of resources and environment. The key to reducing carbon emissions and maintaining economic growth lies in improving carbon productivity, which has become a key measure to achieve low-carbon development and combat climate change.

The world economy is gradually changing to a technology-supported, data-secured, knowledge-led digital economy driven by the new round of global technological revolution and industrial change. The digital economy has become an important engine for improving resource use efficiency, promoting green economic development and enhancing carbon productivity [3]. It has also become an important part of the economies of countries worldwide. According to the China Academy of Information and Communications Technology, in 2021, the digital economy in 47 countries worldwide will be USD 38.1 trillion, accounting for 45% of GDP and representing a nominal growth of 15.6% year-on-year [4]. Among them, China has the second largest digital economy in the world, with USD 7.1 trillion, accounting for 39.8% of GDP and a nominal growth of 16.2% year-on-year [5]. In recent years, the rise of Chinese digital economy enterprises has accelerated, gradually forming digital economy industry clusters led by leading enterprises. The Chinese government is actively building digital economy industrial parks, constructing a digital economy industrial park policy system, and promoting the clustering of key digital economy industries. According to incomplete statistics from the China Academy of Information and Communications Technology, the number of digital economy industrial parks around the country is growing rapidly. As of March 2022, more than 200 industrial parks were named after the “digital economy” [5]. The rapid clustering of digital economy industries has contributed to the improvement in energy efficiency and the rapid development of low-carbon industries, effectively raising carbon productivity.

As an information and communication industry covering communication equipment, application software, and various Internet services, the information and communication technology (ICT) industry is a pillar industry for developing the digital economy [6,7]. The rapid development of the digital economy has led to a concentration of upstream- and downstream-related enterprises, and the ICT manufacturing industry and ICT service industry have formed a pattern of interactive and integrated development. The trend in ICT industrial co-agglomeration is becoming increasingly obvious, which creates opportunities for the further development of a low-carbon economy. The prominent role of the ICT industry in carbon productivity is manifested in two ways. On the one hand, ICT industrial co-agglomeration has a factor reallocation effect, scale effect, cost reduction effect, and knowledge spillover effect, which can promote enterprise technology innovation, improve production and life efficiency, promote industrial upgrading and energy saving and emission reduction, and further improve regional innovation level as well as economic development level, thus improving carbon productivity. On the other hand, in the process of ICT industrial co-agglomeration, ICT equipment manufacturing and operation may produce rebound effect of energy consumption, which is not conducive to energy consumption reduction [8]. With the increasing scale of co-agglomeration, ICT industrial co-agglomeration may produce a crowding effect and increase the pressure of energy conservation and emission reduction [9], so whether ICT industrial co-agglomeration can improve carbon productivity is an urgent issue to be studied. Meanwhile, the differences in geographic location, resource endowment, and economic level among regions in China may make the ICT industrial co-agglomeration development form an uneven economic and geographical pattern [10]. Therefore, it is equally important to study whether there are heterogeneous results on the impact of ICT industrial co-agglomeration on carbon productivity in different regions.

We aimed to verify whether ICT industrial co-agglomeration has a driving effect on improving carbon productivity in the digital economy and regional development imbalance and clarifying the patterns and differences of this effect in time and space. Using China’s provincial panel data from 2009 to 2019, this paper analyzes the spatio-temporal characteristics of ICT industrial co-agglomeration and carbon productivity in China and examines the relationship between ICT industrial co-agglomeration and carbon productivity by adopting a dynamic panel data model. Additionally, it discusses the spatio-temporal heterogeneity of the impact of factors such as ICT industrial co-agglomeration on carbon productivity through a geographically and temporally weighted regression (GTWR) model.

The article is organized next as follows: the second part is a literature review and theoretical hypotheses, the third part is a model setting and description of variables, the fourth part is empirical analysis, and the fifth part is conclusions and policy recommendations.

## 2. Literature Review and Theoretical Hypotheses

### 2.1. Literature Review

#### 2.1.1. Industrial Co-Agglomeration

Industrial co-agglomeration is not only the continuous clustering of a single industry in space but also the joint clustering of related industries. Ellison and Glaeser [11] first move away from research on individual industry agglomeration and focus on the diversity of industry co-agglomeration. They believe industrial co-agglomeration is a spatial agglomeration phenomenon of interconnection between different industries. Scholars have explored the formation mechanism of industrial co-agglomeration from different perspectives. Based on industrial agglomeration theory, Steijn et al. [12] and Diodato et al. [13] argue that the spatial co-agglomeration of different industries is influenced by Marshall externalities (i.e., input–output linkages, skilled labor, and knowledge spillovers). In addition, other scholars Duranton and Overman [14] and Gallagher [15] suggest that mechanisms such as circular causal cumulative effects, policy intervention, transportation costs, and information costs are responsible for the formation of industrial co-agglomeration. Moreover, scholars have carried out much research on the phenomenon of industrial co-agglomeration from a spatial perspective. Ke et al. [16] argue that manufacturing and productive service industries are mutually attracted to each other, co-clustering spatially, and that industrial co-agglomeration has a spillover effect on neighboring cities. Barrios et al. [17] explore the phenomenon of spatial co-agglomeration between domestic and foreign multinational firms using manufacturing data from Ireland. They find that multinational firms positively impact domestic manufacturing firms’ productivity and that the co-agglomeration of domestic and foreign manufacturing firms brought about positive spillover effects.

#### 2.1.2. Carbon Productivity

Carbon productivity is the economic benefit generated per unit of CO_2_ emissions over a while [18]. Currently, in addition to the measurement of carbon productivity indicators, research on carbon productivity has focused on two main areas. First is the study of carbon productivity influencing factors. Scholars mainly focus on the influence of industrial agglomeration, technological progress, environmental regulation, energy structure, industrial structure, FDI, and external openness on carbon productivity [19,20,21]. Second is the study of the spatial and temporal heterogeneity of carbon productivity. Studies on carbon productivity changes in the time dimension have generally concluded that a fluctuating increase over time characterizes China’s carbon productivity [22]. Meanwhile, Sun et al. [23] conclude that 83 countries/regions’ carbon productivity increased slowly from 1990 to 2017. Studies on carbon productivity changes in the spatial dimension have generally concluded that there are spatial differences in carbon productivity, with a decreasing trend in a stepwise manner in the eastern, central, and western regions of China [24,25]. From a world perspective, Bai et al. [26] argue that carbon productivity has generally grown faster in developed countries/regions than in developing countries/regions.

#### 2.1.3. Industrial Co-Agglomeration and Carbon Productivity

The current research on the relationship between industrial co-agglomeration and carbon productivity is mainly in the following two aspects: (1) research on the role of industrial co-agglomeration on economic development. Industry co-agglomeration is a manifestation of industrial development and industrial structure upgrading. Many countries seek a “two-wheel drive” model in which manufacturing and service industries develop together to promote regional economic growth. Lanaspa et al. [27] argue that synergistic effects and co-agglomeration economies between manufacturers and intermediate producer services generate geographical clusters that ultimately contribute to regional economic growth. Li et al. [28] find that co-agglomeration of logistics and manufacturing industries can promote green total factor productivity in local and surrounding areas through economies of scale, the knowledge spillover effect, and the resource allocation effect. (2) Research on the mechanism of the role of industrial co-agglomeration on the environment. Industrial co-agglomeration brings about a scale effect, promotes healthy competition among enterprises and continuously eliminates outdated and highly polluting enterprises. It has facilitated effective environmental management by the government [29]. At the same time, however, industrial coalescence increases the density of economic activity and limited factor inputs in areas where resources are scarce, exacerbating environmental pollution. Scholars have mostly studied environmental pollution from different types of industrial co-agglomeration but have yet to reach a consensus. Fan et al. [30] argue that the coalescence of financial and manufacturing industries harms environmental pollution. Among them, the optimization of industrial structure and the expansion of credit scale contributed to environmental protection. Li et al. [31] believe that the co-agglomeration of producer services and manufacturing can promote carbon intensity reduction in regions with reasonable resource allocation. Based on Chinese provincial panel data from 2004 to 2019, Zhuang et al. [32] analyze a regional difference of “high in the east and low in the west” in the co-agglomeration of effective service and manufacturing industries in China. Additionally, industrial co-agglomeration can significantly reduce air pollution using a spatial econometric model, and the air pollution reduction from industrial co-agglomeration has a significant spatial spillover effect. Yang et al. [33] find that government-led industrial co-agglomeration significantly contributes to environmental pollution management, while market-driven industrial co-agglomeration promotes environmental pollution management in surrounding areas through spatial spillover effects. Additionally, industrial co-agglomeration can improve environmental quality through technological innovation. Other scholars put forward the opposite view, Zhang et al. [34] argue that industrial co-agglomeration aggravates environmental pollution, and there is a significant threshold effect. The relationship between the development of industrial co-agglomeration and environmental pollution shows an inverted S-shaped curve.

In addition, other scholars have explored the impact of the ICT industry on the economy and environment. ICT can implement technological improvements and optimal configurations for traditional industries and has great potential to support the development of a low-carbon economy [35]. Cui et al. [36] argue that different ICT-produced capital stocks have multiple impacts on carbon emissions indirectly through the digital economy and energy efficiency. Sun and Kim [37] conclude that there is a positive contribution of ICT industry development to low-carbon development, which can improve environmental quality. Chatti and Majeed [38] find that the interaction of increasing ICTs and urbanization can reduce carbon emissions and improve environmental quality. However, few scholars have discussed the impact of ICT industry agglomeration on the environment. Based on panel data of the Yangtze River Delta region in China from 2003 to 2016, Wang et al. [39] argue that ICT industry agglomeration significantly increases carbon emissions and negatively affects the environment. Moreover, when technological innovation reaches a certain threshold, ICT industrial agglomeration will significantly reduce carbon emissions.

#### 2.1.4. Literature Review and Marginal Contributions

Based on the above literature review, we find that a large body of literature already argues for the relationship between industrial co-agglomeration and carbon productivity. However, in some areas, further research is still needed: (1) Current research mostly discusses different industry co-location patterns, with most relevant studies starting from the co-location of production services and manufacturing industries. However, fewer scholars have focused on ICT industry clustering, ICT manufacturing and ICT service industry co-location and their impact mechanisms. China’s ICT manufacturing and ICT service industries are developing rapidly, and industrial co-agglomeration patterns are gradually taking shape in the context of government-led and autonomous enterprise development, so ICT industrial co-agglomeration is worth discussing. (2) The importance of promoting low-carbon economic development through industrial co-agglomeration has received increasing attention. The ICT industry is a resource-saving and environmentally friendly industry whose characteristics predict that ICT industrial co-agglomeration development will have an important impact on carbon productivity. However, there currently needs to be more relevant studies. (3) From the literature exploring the influencing factors of carbon productivity, research methods include fixed effects models, multiple regression models, spatial autoregressive models, and spatial Durbin models. However, these research methods cannot effectively respond to the effects of different factors in different regions and need more strength to explain the reality.

Compared with the existing literature, the marginal contributions of this paper are as follows: (1) This paper introduces the discussion of industrial co-agglomeration to two related industries, ICT manufacturing and ICT services, and systematically studies the level of ICT industrial co-agglomeration in China, enriching the existing research results. The use of industrial co-agglomeration indicators can reflect the development of collaboration among ICT industries and provide more refined and comprehensive data support for subsequent studies. (2) It focuses on the important role ICT industrial co-agglomeration plays in regional economic growth and environmental governance. The article systematically examines the relationship between ICT industrial co-agglomeration and carbon productivity, enriching the theoretical study of ICT industrial co-agglomeration to promote green and low-carbon development, broadening the scope of environmental economics research to a certain extent, and providing Chinese experience to countries around the world. (3) Incorporating temporal and spatial information into the research model, the extent of the role of different factors on carbon productivity is systematically explored. In addition, the temporal and spatial heterogeneity of the impact of ICT industrial co-agglomeration on carbon productivity is discussed, providing new perspectives and ideas for future regional differentiated policy formulation and academic research.

### 2.2. Theoretical Hypotheses

In the era of the digital economy, the ICT industry has become the “leading industry” to drive economic growth, which has the characteristics of high technology, low pollution, and low energy consumption and can drive the development of related industries [40]. With ICT manufacturing and service industries showing a clear trend of co-agglomeration in space, it has enormously contributed to economic development and environmental quality improvement. On the one hand, industrial co-agglomeration brings in advanced industries and promotes the re-integration and clustering of factors. The digital economy continues strengthening the links between ICT and local industries, further stimulating the ICT industrial co-agglomeration effect. ICT industrial co-agglomeration achieves intensive and large-scale development through economies of scale [41]. Through positive externalities—sharing labor markets and public infrastructure and reducing the cost of information access—it improves production efficiency, reduces energy consumption, saves transportation costs, and optimizes resource allocation. Therefore, ICT industrial co-agglomeration dampens carbon emissions while promoting incremental returns to the scale of ICT enterprises. He et al. [42] argue that manufacturing agglomeration creates a market demand for intermediate goods, and the supporting productive services are further formed and expanded. As a result, industry coalescence exerts a huge externality effect through industry linkages and circular causal accumulation effects. On the other hand, ICT industrial co-agglomeration enhances knowledge sharing among enterprises and technological advancement through the knowledge spillover effect [43], improves regional innovation, upgrades energy saving and green technology, and thus increases carbon productivity. At the same time, in the digital economy’s background, ICT industrial co-agglomeration can use data and other vital elements to provide technical consultation and services to related industries through the resource-sharing mechanism [44]. Additionally, it can optimize production processes, and improve the efficiency of production, sales, and consumption of products, thus bringing economic benefits to regional development and reducing carbon emissions. Therefore, we propose the following hypothesis:

**Hypothesis 1.**  *ICT industrial co-agglomeration positively affects the increase in carbon productivity.*

Due to the differences in resource endowment, development stage, economic level, environmental quality, industrial structure, and strategic positioning among regions in China [45], the impact of ICT industrial co-agglomeration and other factors on carbon productivity varies in time and space. In the background of the digital economy, ICT is considered the key to resolving regional development imbalances [46]. ICT industrial co-agglomeration can break through geographical constraints, optimize the spatial layout of production factors and promote the coordinated development of China’s low-carbon economy. Developed regions with a better economic foundation can bring into play the strong dynamics of the digital economy by relying on their solid technological level and factor allocation capabilities. It enables ICT industrial co-agglomeration to accelerate industrial upgrading and transformation and reduce carbon productivity through the diffusion and penetration of knowledge. Less economically developed regions such as Western China can make up for their development shortcomings through the digital economy, transforming production and consumption patterns and thus achieving green and low-carbon development. Zafar et al. [47] argue that ICT penetration in remote areas directly impacts economic development and that ICT investment provides new jobs, thereby enhancing regional social living standards. In addition, less economically developed regions take over part of the ICT manufacturing industry, promoting regional technological progress, which drives economic growth and promotes coordinated regional development. Hence, we propose:

**Hypothesis 2.**  *The effect of factors such as ICT industrial co-agglomeration on carbon productivity has significant spatial and temporal heterogeneity. For most regions, ICT industrial co-agglomeration can increase carbon productivity.*

**Figure 1:**
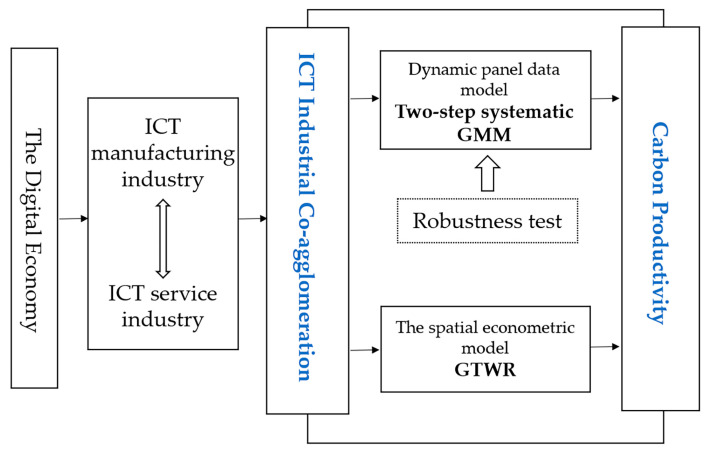
Research Framework.

Based on the above analysis, the research framework of this paper is shown in Figure 1:

## 3. Materials and Methods

### 3.1. Research Sample Selection

In 2009, the Chinese government proposed the “China Awareness” strategy to apply various ICTs to various industries in depth, to specialize and refine the management of production and life. In the same year, the Chinese government decided to promote network convergence and full-service operations, which became crucial in developing the digital economy. This paper selects panel data for 30 Chinese provinces from 2009 to 2019 (data for other provinces are missing) to test the impact of ICT industrial co-agglomeration on carbon productivity in the background of the digital economy. Considering the availability and continuity of data, this paper sets the ICT manufacturing industry as the manufacture of electronic equipment and communication equipment industry in the *China Statistical Yearbook on High Technology Industry* [48]. Moreover, it sets the ICT service industry as the information transmission, software, and information technology service industry in the *China Statistical Yearbook of Tertiary Industry* [49].

### 3.2. Research Method Selection

#### 3.2.1. Dynamic Panel Model

To test the theoretical hypothesis 1 proposed in the previous paper and to examine the effect of ICT industrial co-agglomeration on carbon productivity, this paper uses carbon productivity as the explanatory variable and ICT industrial co-agglomeration as the explanatory variable. Moreover, other factors affecting carbon productivity are introduced into the model as control variables. The econometric model of ICT industrial co-agglomeration and carbon productivity is as follows:

(1) $${\mathrm{cp}}_{i,t}{=\alpha }_{0}{+\alpha }_{1}{\mathrm{cp}}_{i,t-1}{+\alpha }_{2}{\mathrm{coagg}}_{i,t}+\overset{N}{\underset{k=3}{\sum }}\alpha_{k}X_{i,t}{+\varepsilon }_{i,t}$$

In Equation (1), i and t represent the province and time, respectively; ${\mathrm{cp}}_{i,t}$ is carbon productivity, ${\mathrm{coagg}}_{i,t}$ is ICT industrial co-agglomeration, $X_{\mathrm{it}}$ is a set of control variables, and $\varepsilon_{\mathrm{it}}$ is the random disturbance term.

Carbon productivity changes are dynamic and continuous, i.e., carbon productivity in the previous period affects carbon productivity in the current period [50]. In order to reveal the dynamic change characteristics of carbon productivity, this paper incorporates the first-order lag term of the dependent variable into the explanatory variables. The dynamic panel model is set as follows:

(2) $${\mathrm{cp}}_{i,t}{=\alpha }_{0}{+\alpha }_{1}{\mathrm{cp}}_{i,t-1}{+\alpha }_{2}{\mathrm{coagg}}_{i,t}+\overset{N}{\underset{k=3}{\sum }}\alpha_{k}X_{i,t}{+\varepsilon }_{i,t}$$

In Equation (2), ${\mathrm{cp}}_{i,t-1}$ is the lag term of carbon productivity, and the meaning of each other symbol is the same as above.

To address the endogenous problem of the lag-dependent variable in the dynamic equation and possibly reduce the interference of omitted variables and measurement errors in the estimation results, this paper uses a two-step approach to estimate a systematic GMM dynamic unbalanced panel model. It uses the first-order lagged term of carbon productivity as an instrumental variable in the difference equation. The system GMM model requires two tests: one is the Arellano–Bond autocorrelation test. It is used to test whether there is a second-order serial correlation in the residuals in the difference equation. The model is set correctly if there is no second-order serial correlation (*p*-Value of AR(2) greater than 0.1). The other is an overidentification test to detect whether the lag term is valid as an instrumental variable. It needs to pass the Hansen test. Moreover, when the *p*-Value of the Hansen test is greater than 0.1, it indicates that the instrumental variable set by the systematic GMM model is valid.

#### 3.2.2. Geographically and Temporally Weighted Regression Model (GTWR)

The traditional global regression model OLS (ordinary least squares) can only estimate the average for the whole sample, which cannot reflect the heterogeneity of coefficients in different regions and cannot effectively explore the local idiosyncrasies among variables. In order to fully reflect the heterogeneous information of different regions and further examine the spatial and temporal differences in the effects of variables such as ICT industrial co-agglomeration on carbon productivity, this paper uses the GTWR model to measure the influence factors of carbon productivity across regions at different times. Geographically weighted regression (GWR) can effectively address the spatial heterogeneity of carbon productivity in the regression problem, but it cannot adequately consider the trend in the time dimension. The GTWR model enables the analysis of impact factors in different spatio-temporal dimensions and provides an important tool for analyzing spatio-temporal non-stationarity of regression coefficients for each variable [51,52]. The formula is as follows:

(3) $$y_{i}{=\beta }_{0}\left(u_{i}{,v}_{i}{,t}_{i}\right)+\sum_{k=1}^{p}\beta_{k}\left(u_{i}{,v}_{i}{,t}_{i}\right)Z_{\mathrm{ik}}{+\tau }_{i}$$

In Equation (3), $\left(u_{i}{,v}_{i}{,t}_{i}\right)$ is the spatio-temporal coordinate of the ith sample point, $\beta_{0}\left(u_{i}{,v}_{i}{,t}_{i}\right)$ denotes the regression constant of the ith sample point, i.e., the constant term in the model, $Z_{\mathrm{ik}}$ is the value of the kth explanatory variable at point i, $\tau_{i}$ is the random error term, and $\beta_{k}\left(u_{i}{,v}_{i}{,t}_{i}\right)$ is the kth regression coefficient of the ith sample point, which is estimated as follows:

(4) $$\hat{\beta }\left(u_{i}{,v}_{i}{,t}_{i}\right)={\left[X^{T}W\left(u_{i}{,v}_{i}{,t}_{i}\right)X\right]}^{-1}X^{T}W\left(u_{i}{,v}_{i}{,t}_{i}\right)Y$$

In Equation (4), $\hat{\beta }\left(u_{i}{,v}_{i}{,t}_{i}\right)$ is the estimated value of $\beta_{k}\left(u_{i}{,v}_{i}{,t}_{i}\right)$, X is the matrix composed of explanatory variables, Y is the matrix composed in the sample, and $W\left(u_{i}{,v}_{i}{,t}_{i}\right)$ is the matrix of spatio-temporal weights. This study draws on the approach of Huang et al. [53] and is based on adaptive bandwidth, Gaussian kernel function and Euclidean distance construction, and is determined using the AICc rule. Based on the theoretical model of GTWR, we will further investigate the spatio-temporal heterogeneity of factors influencing carbon productivity in China with the following equations.

(5) $${\mathrm{cp}}_{i}{=\gamma }_{0}\left(u_{i}{,v}_{i}{,t}_{i}\right)+\sum_{k=1}^{p}\gamma_{k}\left(u_{i}{,v}_{i}{,t}_{i}\right)Z_{i,k}{+\theta }_{i,t}$$

In Equation (5), $\left(u_{i}{,v}_{i}{,t}_{i}\right)$ is the spatio-temporal coordinates of province, $\gamma_{k}\left(u_{i}{,v}_{i}{,t}_{i}\right)$ is the regression coefficient, $Z_{i,k}$ is a set of explanatory variables, $\gamma_{0}\left(u_{i}{,v}_{i}{,t}_{i}\right)$ is the constant term, $\theta_{\mathrm{it}}\;$is the random disturbance term.

### 3.3. Variables Selection

#### 3.3.1. Explained Variable: Carbon Productivity (cp)

This paper uses the ratio of regional GDP to CO_2_ emissions to measure carbon productivity. Among them, the GDP data of each region are deflated with 2009 as the base period, and CO_2_ emissions are calculated according to the emission accounting method provided by the Intergovernmental Panel on Climate Change (IPCC) [54].

#### 3.3.2. Core Explanatory Variable: ICT Industrial Co-Agglomeration (Coagg)

The current indicators for measuring industrial co-agglomeration include the E g index constructed by Ellison, Glaeser [11], and others and the D-O index constructed by Duranton and Overman [14]. The D-O index is more limited by the data and less practical, so this paper selects the modified EG index to measure the ICT industrial co-agglomeration by referring to the measures of Zheng and He [55]. The calculation formula is as follows:

(6) $${\mathrm{coagg}}_{\mathrm{it}}=\left(1-\frac{{|\mathrm{Magg}}_{\mathrm{it}}{-\mathrm{Sagg}}_{\mathrm{it}}|}{{\mathrm{Magg}}_{\mathrm{it}}{+\mathrm{Sagg}}_{\mathrm{it}}}\right){+\mathrm{Magg}}_{\mathrm{it}}{+\mathrm{Sagg}}_{\mathrm{it}}$$

Among them, ${\mathrm{Magg}}_{\mathrm{it}}$ and ${\mathrm{Sagg}}_{\mathrm{it}}$ are the location entropy of ICT manufacturing and ICT service industries, respectively, to measure the industrial agglomeration, which is calculated by the number of employees in each industry. The larger the value of ${\mathrm{coagg}}_{\mathrm{it}}$, the stronger the interdependence and correlation between the region’s ICT industries, and the higher the level of co-agglomeration. Otherwise, the situation is the opposite. The modified E g index selected in this paper measures the co-agglomeration quality and co-agglomeration level from the perspective of industrial co-development from microscopic enterprise data. It can reflect the spatial agglomeration status between ICT manufacturing and ICT service industries in a more comprehensive way.

#### 3.3.3. Control Variables

(1) Degree of external openness (open): External openness facilitates the optimization of regional resource allocation. Zhang et al. [56] and Shahbaz et al. [57] argue that external openness can increase domestic technological progress, and in turn, this degree significantly impacts local carbon emissions and economic development. This paper selects the ratio of total import and export and GDP in each region to indicate the degree of external openness. The relevant data are converted at the exchange rate of the calendar year.

(2) Industrial structure (instr): The industrialization process promotes rapid economic development but also causes various types of environmental pollution. However, optimizing the industrial structure and promoting innovation-driven development of industries can effectively improve carbon productivity [58]. This paper selects the ratio of value added of the secondary industry and GDP in each region to measure the industrial structure.

(3) Energy consumption structure (enstr): Coal is a highly polluting and emitting energy source. Li et al. [59] and Xiao et al. [60] propose that optimizing the energy consumption structure with coal as the mainstay is conducive to achieving energy conservation, emission reduction, and green development. This paper chooses the ratio of coal consumption to total energy consumption in each region to represent the energy consumption structure. The relevant data are converted into standard coal.

(4) Environmental regulations (er): Implementing environmental regulations can reinforce the emission reduction effect of industrial co-agglomeration. Pei et al. [61] and Shang et al. [62] argue that reasonable environmental regulations can promote economic development while forcing enterprises to improve green technology and reduce carbon emissions. This paper selects the ratio of a completed investment in industrial pollution control to GDP of each region to represent the intensity of environmental regulation in each region.

### 3.4. Data Source

To ensure the consistency of statistical caliber, the data were mainly obtained from *China Statistical Yearbook on High Technology Industry* [48], *China Statistical Yearbook of Tertiary Industry* [49], *China Energy Statistical Yearbook* [63], *China Statistical Yearbook* [64], and the China Stock Market & Accounting Research Database (CSMAR) [65]. Moreover, some missing data were completed by interpolation. Table 1 shows the descriptive analysis of the above variables.

## 4. Results

### 4.1. Spatio-Temporal Variation Analysis of ICT Industrial Co-Agglomeration and Carbon Productivity

**Figure 2:**
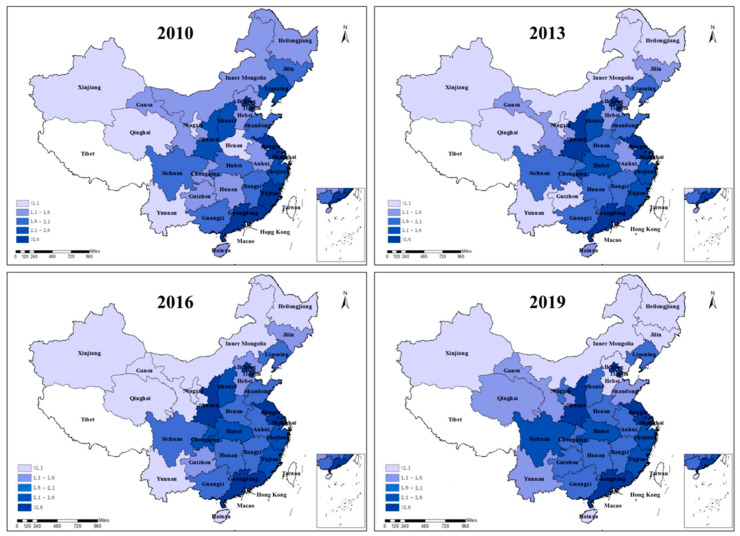
Spatio-temporal variation in ICT industrial co-agglomeration in China (2010, 2013, 2016, and 2019).

In order to explore the spatial distribution and evolution process of ICT industrial co-agglomeration in China, this paper uses ArcGIS 10.2 software to map the ICT industrial co-agglomeration in 30 provinces of China using 2010, 2013, 2016, and 2019 as nodes (in Figure 2).

As shown in Figure 2, the darker the color in the graph, the higher the degree of ICT industrial co-agglomeration in the region. It can be seen that from 2010 to 2019, China’s ICT industrial co-agglomeration, in general, shows an upward trend, and there are significant regional differences, showing the characteristics of “high in the east and low in the west”. Specifically, (1) the eastern region has a higher degree of ICT industrial co-agglomeration, with Beijing, Tianjin, Jiangsu, Shanghai, and Guangdong continuing to lead in ICT industrial co-agglomeration. (2) Driven by the effect of the eastern region and the influence of industrial transfer, the central-region ICT industrial co-agglomeration since 2010 enhances the apparent effect. (3) The ICT industrial co-agglomeration degree in the western region is low. However, the ICT industrial co-agglomeration degree in Sichuan Province, Shaanxi Province, and Guangxi Zhuang Autonomous Region has been steadily higher compared to other provinces in the western region. (4) The ICT industrial co-agglomeration degree in Northeast China has decreased since 2010. In general, ICT industrial co-agglomeration in the central and western regions has improved since 2010. The reason is that the level of ICT industrial co-agglomeration in the eastern region is in a leading position in China, and the central region is radiated and driven by the eastern region, which improves the level of ICT industrial co-agglomeration. In addition, the Chinese government continues to optimize the allocation of resources in the central and western regions, improve the relevant institutional construction, guide the ICT manufacturing industry in the central and western regions, drive the information construction in the central and western regions, and promote coordinated regional development. Therefore, the ICT industrial co-agglomeration degree in some provinces in the central and western regions has improved significantly.

**Figure 3:**
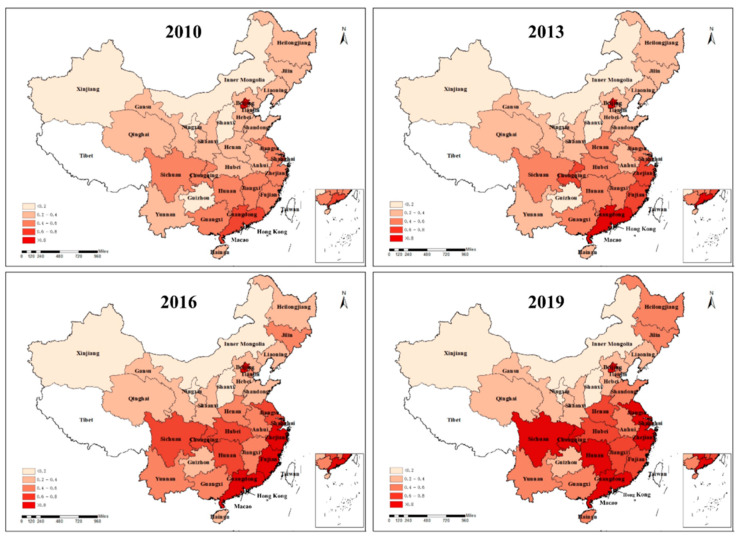
Spatio-temporal variation in carbon productivity in China (2010, 2013, 2016, and 2019).

In order to explore the spatial distribution and evolution process of carbon productivity in China, this part uses ArcGIS 10.2 software to map the carbon productivity in 30 provinces of China using 2010, 2013, 2016, and 2019 as nodes (in Figure 3).

As shown in Figure 3, the darker color in the graph represents the higher carbon productivity of the region. It can be seen that there are significant regional differences in China’s carbon productivity over the sample period, with similar “high in the east and low in the west” characteristics as the ICT industrial co-agglomeration. Specifically, (1) the Eastern region has higher carbon productivity, with Beijing continuing to lead. (2) Except for Shanxi Province, where the change in carbon productivity is insignificant, all other provinces in the central region significantly increase carbon productivity. (3) The carbon productivity in the western region is low, and only Sichuan Province and Chongqing City have a significant carbon productivity enhancement effect, which indicates that the western region has more room for carbon emission reduction and economic development. (4) Carbon productivity in the northeast improves after 2013. In general, China’s carbon productivity shows an upward trend, which indicates that China continues to pay attention to environmental issues while focusing on economic development and constantly seeks a balance between economic growth and resources and the environment. Meanwhile, some Chinese provinces have persistently low carbon productivity, probably because they are primarily coal-producing regions (e.g., Shanxi Province, Shaanxi Province, and Inner Mongolia Autonomous Region), and have insufficient incentives to transform and upgrade their industries. In addition, the long-term crude expansion of growth and inefficient utilization of coal resources makes it difficult to solve the environmental pollution problem. These areas in the future also need to accelerate the development of green, low-carbon transformation.

### 4.2. Results of GMM

The results of the GMM test are shown in Table 2, and the *p*-Values for AR(2) are all larger than 0.1, indicating that the estimated results pass the autocorrelation test. In addition, the Hansen J statistics correspond to *p*-Values above 0.1, indicating that the instrumental variables selected in this paper are reasonable. Column (1) of Table 2 includes only the first-order lag term of carbon productivity and the core explanatory variables. The regression coefficient of ICT industrial co-agglomeration is significantly positive, indicating that ICT industrial co-agglomeration has a catalytic effect on carbon productivity, which confirms Hypothesis 1 and some scholars’ views. Ye et al. [66] also argue that industrial co-agglomeration reduces air pollutant emissions through technological advances. In addition, the conclusions of this paper are similar to those of Yan et al. [67] They argue that the digital economy can indirectly reduce carbon emission intensity through the ICT industry channel. After adding the control variables, i.e., Columns (2)–(5) of Table 2, the regression coefficients of ICT industrial co-agglomeration are still significantly positive at the 1% significance, with only a slight change in the coefficients, indicating that the regression results are relatively robust. ICT industrial co-agglomeration does have a catalytic effect on low-carbon economic development. With the development of the digital economy, ICT industrial co-agglomeration can optimize the division of labor, strengthen exchanges and cooperation among enterprises, and expand the advantages of technological progress. Meanwhile, ICT industrial co-agglomeration brings sustainable development opportunities to traditional industries, improves the green performance of traditional industrial production [68], and thus increases carbon productivity.

The regression coefficients of the first-order lag term of carbon productivity are all significantly positive, indicating a virtuous cycle between carbon productivity in the previous period and the current period. The regression coefficient of the degree of external openness is significantly negative, indicating that external openness does not lead to an increase in carbon productivity, because increasing the scale of import and export trade and introducing labor-intensive enterprises with low value-added and low-technology content further increase the pressure on energy conservation and emission reduction, thus inhibiting the improvement in carbon productivity. The regression coefficients of the industrial structure are all significantly negative. The secondary industry is the primary source of carbon emissions, and the larger its share in the industrial structure, the more unfavorable it is to improving carbon productivity. The regression coefficient of energy consumption structure is significantly negative, indicating that increasing coal-based energy consumption inhibits the improvement in carbon productivity. As a traditional energy source, coal is one of the crucial sources of carbon emissions, and the increase in its consumption share will inhibit green and low-carbon economic development. The insignificant effect of environmental regulations on carbon productivity maybe since environmental regulations have not been effective in pushing energy-intensive firms to reform and thus play an energy-saving and emission-reducing role.

### 4.3. Robustness Test

To further ensure the reliability of the experimental results, this paper performs robustness tests in terms of replacing core explanatory variable measures and sample time groupings.

#### 4.3.1. Robustness Tests based on Other Measures of ICT Industrial Co-Agglomeration

The first robustness test uses the method of replacing the core explanatory variables. Considering the consistency and availability of data, this paper replaces the number of employees with the number of enterprises [69,70] in ICT manufacturing and ICT services in each region to calculate the location entropy and replace the indicator measuring the ICT industrial co-agglomeration. The test continues to use the two-step systematic GMM model, as shown in Table 3, and the sign direction and significance of the core explanatory variables are consistent with the above empirical results, proving that the findings of this paper are robust.

#### 4.3.2. Robustness Tests based on Different Time Periods

The second robustness test re-estimates the sample into two periods, 2009–2014 and 2014–2019. The explanatory variables, explained variables, estimation methods and tests remain unchanged except for changes in the period. The estimation results are shown in Table 4. The regression results obtained from the analysis of subsamples introducing different time horizons are similar to the empirical results in the previous paper. The systematic GMM models for both time horizons pass the AR test and Hansen test, and the estimated coefficients of the core explanatory variables remain consistent and significant in the same direction. The results of the robustness test introducing different time horizons again prove the robustness of the results of this paper.

### 4.4. Results of GTWR

The above results show that ICT industrial co-agglomeration is essential to sustainable regional development. Based on the fact that different regions in China differ in resource endowment, economic level, industrial structure, and policy implementation, this section examines whether there is spatio-temporal heterogeneity in the role of ICT industrial co-agglomeration and other factors in influencing carbon productivity. Based on the provincial and regional panel data from 2009 to 2019, this paper regressed Formula (5) using ArcGIS 10.2 software. The GTWR regression results are shown in Table 5. In addition, a visual representation of the ICT industrial co-agglomeration regression coefficients was performed with ArcGIS 10.2 software to demonstrate the impact of ICT industrial co-agglomeration regression coefficients on carbon productivity in both time and space dimensions. The GTWR model regression results have an R2-value of 0.972 and an AICc value of −754.417, which indicates that the GTWR fit is relatively significant.

**Figure 4:**
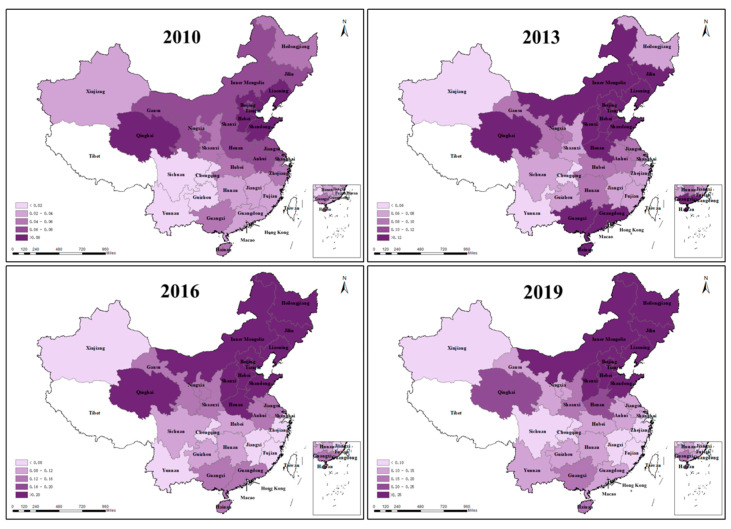
Spatial and temporal variation in regression coefficients of ICT industrial co-agglomeration (2010, 2013, 2016 and 2019).

As can be seen in Figure 4, there are significant temporal and spatial differences in the regression coefficients of ICT industrial co-agglomeration, and the impact of ICT industrial co-agglomeration on carbon productivity increases as time advances. In 2010, the regression coefficients of ICT industrial co-agglomeration generally lie between 0.02 and 0.08, and the values grow to between 0.1 and 0.25 in 2019, and the degree of impact is greater in the northern regions. As the degree of ICT industrial co-agglomeration continues to increase, its positive impact on carbon productivity at the national level deepens. As can be seen from Table 4, the average regression coefficient for ICT industrial co-agglomeration was 0.11716, ranging from −0.00143 to 0.40248, indicating that ICT industrial co-agglomeration contributed positively to carbon productivity in most regions and years during the study period, consistent with the results shown in the baseline regression. This empirical result reflects the positive impact on carbon productivity despite the significant differences in the degree of ICT industrial co-agglomeration across regions. It has similarities with the findings of Zhang et al. [71] They concluded that for 90% of the regions in China, industrial co-agglomeration is beneficial in reducing carbon emissions from manufacturing. In contrast to the findings of this paper, Wang et al. [72] concluded that there are regional differences in the impact of the digital economy on low-carbon, sustainable urban development. It may be due to the digital divide with different regional ICT levels. Moreover, the regional digital economy level must fully represent the ICT industrial co-agglomeration level. The positive upper quartile of the degree of external openness indicates that higher external openness in some regions is conducive to increased carbon productivity. With the degree of external openness, some regions actively introduce advanced foreign technology and equipment, the trade structure has been optimized, and enterprise production management methods have been improved, thus increasing economic benefits while reducing the environmental pollution. The average regression coefficient of the industrial structure and the upper quartile are positive, which indicates that as China continues to industrialize, the internal structure of the secondary industry in some regions is constantly being restructured, which has a positive impact on carbon productivity. The coefficient of energy structure is mainly negative, which indicates that optimizing the energy consumption structure and improving energy utilization efficiency is urgent. Environmental regulation is dominated by negative values, which suggests that an increase in the intensity of environmental regulations may be detrimental to the improvement in carbon productivity. China’s industrial pollution control investment may be inefficient, and it is challenging to promote low-carbon economic transformation by overemphasizing the increase in completed investments without changing the investment structure.

## 5. Conclusions and Discussion

### 5.1. Main Conclusions

The ICT industry is advancing smoothly, providing a wealth of digital technologies, products, and services for all sectors of China’s national economy and becoming an endless source of power for the rapid development of the digital economy. In the era of the digital economy, ICT industrial co-agglomeration achieves a win–win situation of economic growth and environmental protection through resource sharing, economies of scale, knowledge dissemination, and penetration. Based on 2009–2019 panel data from 30 provinces in China, this paper analyses the spatial and temporal characteristics of ICT industrial co-agglomeration and carbon productivity and empirically investigates the impact of ICT industrial co-agglomeration on carbon productivity in China by constructing a two-step systematic GMM model. Finally, it examines the spatial and temporal heterogeneity of the factors influencing carbon productivity using the GTWR model and draws the following conclusions: (1) China’s ICT industrial co-agglomeration and carbon productivity show an upward trend during the study period. The regional distribution of ICT industrial co-agglomeration and carbon productivity is similar to China’s regional economic development level, with the characteristics of “high in the east and low in the west”. (2) In China, the development of ICT industrial co-agglomeration can improve carbon productivity, i.e., the ICT service industry and ICT manufacturing industry can be developed in a synergistic and clustered manner to promote healthy competition among ICT enterprises, facilitate industrial upgrading and transformation, and ultimately achieve low-carbon development. (3) The industrial structure with a high share of secondary industry, a coal-based energy consumption structure, and a higher degree of external openness significantly impacts carbon productivity. (4) The effect of ICT industrial co-agglomeration on carbon productivity shows significant spatial and temporal heterogeneity, and the effect increases over time. Although there are significant differences in the degree of ICT industrial co-agglomeration across China, in most regions, ICT industrial co-agglomeration positively impacts carbon productivity.

### 5.2. Policy Implications

The above findings suggest important policy implications.

(1) Deepen the development of the digital economy. First, strengthen the application of the digital economy. Use digital platforms to integrate industrial resources and promote the integration and development of the ICT industry and traditional industries. Strengthen refined production and intelligent management, and promote traditional industries’ digitalization and green transformation. Second, by strengthening new infrastructure construction, policymakers can help develop the digital economy, actively promote the coordinated co-agglomeration of ICT manufacturing and service industries, and drive ICT industry development. Third, enhance the development of the ICT industry. Make up for the shortcomings of the ICT manufacturing industry. Increase the R&D efforts of the ICT manufacturing industry, improve the level of green technology innovation and realize the upgrade of the value chain. In addition, utilize the advantages of the ICT service industry. Take advantage of data resources and the scale of the Chinese market to fully exploit the diversified market needs of the ICT service industry and enhance its competitiveness of the ICT industry. (2) Rational layout of ICT industrial co-agglomeration to promote the development of the low-carbon economy. First, policymakers can introduce and cultivate supporting enterprises around ICT leading enterprises to build a synergistic development pattern of ICT industry and promote the development of the whole chain of ICT industry. At the same time, by accelerating the improvement in co-agglomeration area science and technology finance, security system, support ICT enterprises green technology key research and development projects, the construction of green supply chain management system. Guide ICT enterprises to strengthen R&D cooperation, promote innovation resource sharing, and optimize the innovation environment. Second, through the path of capital financing, technology flow, talent flow, etc., using digital, intelligent, green means to promote the development of ICT industry clusters. Third, policymakers should continue to promote a collaborative industrial development mechanism guided by ICT manufacturing and supported by the ICT service industry to optimize the layout of industrial co-agglomeration, reduce production costs, improve production efficiency, and promote green and sustainable development.

(3) Improve carbon productivity by expanding opening to the outside world, promoting industrial upgrading, optimizing energy consumption structure, and improving investment efficiency in industrial pollution control. First, policymakers should continuously improve the foreign investment access systems, improve the quality of investment attraction, and expand the international division of labor, the field, and the scope of cooperation. Additionally, introduce advanced foreign, clean, low-carbon production technology, and accelerate domestic technological progress. Second, strictly control the scale of production capacity of high energy-consuming and high-emission industries, and promote the optimization and upgrading of industrial structure. Promote R&D and application of low-carbon technologies in secondary industries to improve production efficiency and increase the added value of products, thereby reducing the environmental burden. Third, enterprises should optimize the energy consumption structure, improve energy utilization efficiency, and actively develop and use renewable energy. Encourage public participation in environmental governance and advocate low-carbon green lifestyles and consumption patterns. Fourth, the local government should further adjust the direction and structure of investment in industrial pollution control and encourage enterprises to improve the level of green technology to play the role of environmental regulation fully. Improve laws and regulations related to environmental governance, and use a variety of environmental regulation tools such as emission fee levies, carbon emission rights, emission rights, energy use rights and environmental taxes to improve regional carbon productivity levels.

(4) For China, different regions should fully use their resource endowments and location conditions to reasonably lay out the synergistic development of the ICT industry. Policymakers should further strengthen the ICT industry base in the central and western regions and help ICT manufacturing industries move to the west. Additionally, tilt quality human resources, financial support, and development approaches to the central and western regions to promote the steady improvement in carbon productivity. Use the digital economy to compensate for the shortcomings in development, optimize the industrial undertaking environment, and create an ICT manufacturing cluster ecosystem. In addition, policymakers should promote the transformation and upgrading of traditional industries in Northeast China and cultivate several new ICT industry growth points. For the eastern region, policymakers should continue to play a leading role in ICT industrial co-agglomeration, break through technical barriers and enhance ICT core competitiveness. Focus on energy conservation and emission reduction while economic development, and improve energy utilization efficiency. For countries worldwide, policymakers should give full play to the driving role of digital economy development, promote the flow of production factors, realize green technological innovation and promote the sustainable development of traditional industries. At the same time, the integration of ICT manufacturing and ICT service industries should be promoted, and the role of ICT industrial co-agglomeration on the low-carbon economy should be fully grasped as a channel to accelerate the realization of low-carbon development goals and promote global green development by optimizing the spatial layout of industries.

### 5.3. Research Perspectives

This research may have some limitations that will be addressed in future research. First, limited to the article’s length, this paper focuses on the relationship between ICT industrial co-agglomeration and carbon productivity. Moreover, it further analyzed the spatial and temporal heterogeneity of ICT industrial co-agglomeration affecting carbon productivity without yet exploring the impact mechanism in detail. Therefore, we will examine the mechanisms by which ICT industrial co-agglomeration affects carbon productivity in future studies. Second, existing research divides industrial co-agglomeration into government-led and market-driven. In the subsequent research, we will further investigate the impact mechanisms of different types of ICT industrial co-agglomeration on carbon productivity and the heterogeneity of the impact of different types of ICT industrial co-agglomeration on carbon productivity in different regions to deal with the relationship between government and market in ICT industrial co-agglomeration and promote the development of China’s low-carbon economy.

## Figures and Tables

**Table 1:** Descriptive statistics.

Variable	Obs.	Mean	Std. Dev.	Min	Max
coagg	330	2.213	1.700	0.504	10.28
cp	330	0.477	0.332	0.0850	2.342
open	330	0.283	0.309	0.0130	1.464
instr	330	0.409	0.0899	0.160	0.620
enstr	330	0.672	0.304	0.0180	1.758
er	330	0.128	0.124	0.00210	0.992

**Table 2:** Results of benchmark regression.

Variable	(1)	(2)	(3)	(4)	(5)
${\mathrm{cp}}_{i,t-1}$	1.028 ***	0.991 ***	0.962 ***	0.921 ***	0.921 ***
	(51.66)	(31.78)	(28.28)	(19.33)	(16.83)
coagg	0.02 ***	0.044 ***	0.047 ***	0.042 ***	0.042 **
	(2.7)	(3.39)	(2.67)	(2.61)	(2.34)
open		−0.125 ***	−0.132 ***	−0.138 ***	−0.134 ***
		(−3.22)	(−2.87)	(−3.01)	(−2.61)
instr			−0.172 **	−0.192 ***	−0.196 ***
			(−2.33)	(−3.06)	(−3.00)
enstr				−0.098 **	−0.096 *
				(−2.37)	(−1.78)
er					0.021
					(0.53)
Constant	−0.03 ***	−0.03 *	0.049 *	0.153 ***	0.148 ***
	(−2.72)	(−1.82)	(1.82)	(3.26)	(2.58)
AR(1)	0.014	0.018	0.016	0.018	0.017
AR(2)	0.289	0.246	0.215	0.195	0.186
Hansen test	0.107	0.114	0.183	0.24	0.248
*** p < 0.1, ** p < 0.05, * p < 0.01					

**Table 3:** Robustness results.

Variable	(6)
${\mathrm{cp}}_{i,t-1}$	1.019 ***
	(37.84)
coagg_e_	0.021 **
	(2.1)
open	−0.011
	(−0.51)
instr	−0.194 ***
	(−3.59)
enstr	−0.056
	(−1.41)
er	0.051
	(1.07)
Constant	0.084
	(1.51)
AR(1)	0.012
AR(2)	0.248
Hansen test	0.187
*** p < 0.1, ** p < 0.05	

**Table 4:** Regression results 2.

Variable	2009–2014	2015–2019
${\mathrm{cp}}_{i,t-1}$	0.969 ***	0.963 ***
	(20.12)	(23.89)
coagg	0.046 **	0.044 ***
	(2.02)	(3.15)
open	−0.200 **	−0.208 **
	(−2.20)	(−2.57)
instr	−0.217 ***	−0.289
	(−2.83)	(−1.53)
enstr	−0.116 **	−0.067
	(−2.40)	(−1.48)
er	0.375	−0.167
	(0.74)	(−0.18)
Constant	0.164 ***	−0.147 **
	(2.95)	(2.38)
AR(1)	0.068	0.136
AR(2)	0.339	0.392
Hansen test	0.452	0.477
*** p < 0.1, ** p < 0.05		

**Table 5:** Results of GTWR model.

Variables	Average Value	Minimum Value	Lower Quartile	Upper Quartile	Maximum Value
${\mathrm{COAGG}}_{i,t}$	0.11716	−0.00143	0.06171	0.15153	0.40248
${\mathrm{OPEN}}_{i,t}$	−0.01560	−0.88556	−0.28502	0.12622	2.91869
${\mathrm{INSTR}}_{i,t}$	0.17799	−2.10779	−0.21501	0.54743	1.61105
${\mathrm{ENSTR}}_{i,t}$	−0.38821	−1.03175	−0.61554	−0.28430	1.38679
${\mathrm{ER}}_{i,t}$	−0.31216	−1.76056	−0.52440	−0.06792	0.42783
**Bandwidth:** 0.114996		**AICc:** −754.417		$R^{2}$: 0.971988	

## Data Availability

Publicly available datasets were analyzed in this study. All data come from the *China Statistical Yearbook on High Technology Industry, China Statistical Yearbook of Tertiary Industry, China Energy Statistical Yearbook, China Statistical Yearbook*, and CSMAR.
